# Gadolinium-based coronary CT angiography on a clinical photon-counting-detector system: a dynamic circulating phantom study

**DOI:** 10.1186/s41747-024-00501-w

**Published:** 2024-10-18

**Authors:** Dmitrij Kravchenko, Chiara Gnasso, U. Joseph Schoepf, Milan Vecsey-Nagy, Giuseppe Tremamunno, Jim O’Doherty, Andrew Zhang, Julian A. Luetkens, Daniel Kuetting, Ulrike Attenberger, Bernhard Schmidt, Akos Varga-Szemes, Tilman Emrich

**Affiliations:** 1 Division of Cardiovascular Imaging, Department of Radiology and Radiological Science, Medical University of South Carolina, Charleston, SC USA; 2 Department of Diagnostic and Interventional Radiology, University Hospital Bonn, Bonn, Germany; 3 Quantitative Imaging Laboratory Bonn (QILaB), Bonn, Germany; 4 Clinical and Experimental Radiology Unit, Experimental Imaging Center, IRCCS San Raffaele Scientific Institute, Milan, Italy; 5 School of Medicine, Vita-Salute San Raffaele University, Milan, Italy; 6 Cardiovascular Imaging Research Group, Heart and Vascular Center, Semmelweis University, Budapest, Hungary; 7 Department of Medical Surgical Sciences and Translational Medicine, Sapienza University of Rome—Radiology Unit—Sant’Andrea University Hospital, Rome, Italy; 8 Siemens Medical Solutions USA Inc, Malvern, PA USA; 9 Siemens Medical Solutions, Forchheim, Germany; 10 Department of Diagnostic and Interventional Radiology, University Medical Center of the Johannes Gutenberg-University, Mainz, Germany; 11 German Centre for Cardiovascular Research, Partner Site Rhine-Main, Mainz, Germany

**Keywords:** Aorta (thoracic), Computed tomography angiography, Contrast media, Coronary vessels, Gadolinium-DTPA

## Abstract

**Background:**

Coronary computed tomography angiography (CCTA) offers non-invasive diagnostics of the coronary arteries. Vessel evaluation requires the administration of intravenous contrast. The purpose of this study was to evaluate the utility of gadolinium-based contrast agent (GBCA) as an alternative to iodinated contrast for CCTA on a first-generation clinical dual-source photon-counting-detector (PCD)-CT system.

**Methods:**

A dynamic circulating phantom containing a three-dimensional-printed model of the thoracic aorta and the coronary arteries were used to evaluate injection protocols using gadopentetate dimeglumine at 50%, 100%, 150%, and 200% of the maximum approved clinical dose (0.3 mmol/kg). Virtual monoenergetic image (VMI) reconstructions ranging from 40 keV to 100 keV with 5 keV increments were generated on a PCD-CT. Contrast-to-noise ratio (CNR) was calculated from attenuations measured in the aorta and coronary arteries and noise measured in the background tissue. Attenuation of at least 350 HU was deemed as diagnostic.

**Results:**

The highest coronary attenuation (441 ± 23 HU, mean ± standard deviation) and CNR (29.5 ± 1.5) was achieved at 40 keV and at the highest GBCA dose (200%). There was a systematic decline of attenuation and CNR with higher keV reconstructions and lower GBCA doses. Only reconstructions at 40 and 45 keV at 200% and 40 keV at 150% GBCA dose demonstrated sufficient attenuation above 350 HU.

**Conclusion:**

Current PCD-CT protocols and settings are unsuitable for the use of GBCA for CCTA at clinically approved doses. Future advances to the PCD-CT system including a 4-threshold mode, as well as multi-material decomposition may add new opportunities for k-edge imaging of GBCA.

**Relevance statement:**

Patients allergic to iodine-based contrast media and the future of multicontrast CT examinations would benefit greatly from alternative contrast media, but the utility of GBCA for coronary photon-counting-dector-CT angiography remains limited without further optimization of protocols and scanner settings.

**Key Points:**

- GBCA-enhanced coronary PCD-CT angiography is not feasible at clinically approved doses.
- GBCAs have potential applications for the visualization of larger vessels, such as the aorta, on PCD-CT angiography.
- Higher GBCA doses and lower keV reconstructions achieved higher attenuation values and CNR.

**Graphical Abstract:**

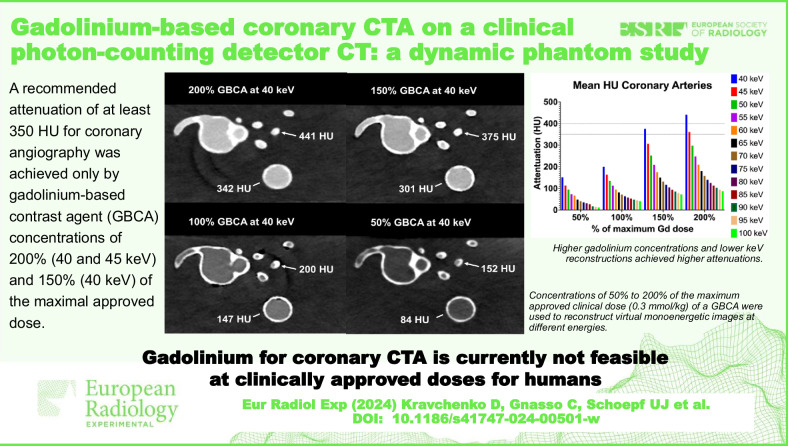

## Background

Coronary computed tomography angiography (CCTA) is a noninvasive diagnostic tool for the assessment of coronary artery disease and has been incorporated in multiple national guidelines over the last decade [1–3]. Adequate visual assessment of coronary vessels requires intravenous injection of contrast media at sufficient concentrations. The most commonly used contrast agents approved for clinical imaging are iodinated contrast media (ICM) for computed tomography (CT) and gadolinium-based contrast agents (GBCAs) for magnetic resonance imaging. The use of ICMs in patients with hyperthyroidism, known iodine allergy, or severely impaired renal function carries certain risks such as anaphylactic shock or contrast-induced nephropathy [4]. This necessitates viable alternatives to ICMs in CT imaging for these purposes. Additionally, having another contrast agent for CT would allow multicontrast imaging on compatible scanners [5]. Historically, the use of GBCA for CT imaging has been limited by the required injected doses and local concentrations to achieve diagnostic attenuation [6]. The introduction of dual-energy CT (DECT) systems allowed the acquisition of images at differing energies facilitating material decomposition, in theory enabling the use of GBCA as a CT contrast at approved dosages, albeit with mixed results [7–9].

Recently, a photon-counting-detector CT (PCD-CT) system has been approved for clinical use. PCD-CT offers significant advantages over conventional energy-integrating-detector CT including reduced electronic noise, improved spatial resolution, a potential reduction in radiation dose, and the ability to acquire spectral data with high temporal resolution [10]. X-ray tubes generate x-rays on a spectrum of energy levels: in energy-integrating-detector CTs, these are integrated to produce a final polyenergetic image. Conversely, using PCD-CT, x-rays can be detected according to their energies in the detector, into so-called ‘bins’ allowing the separation of photon energies [11]. Such binning allows the routine acquisition of spectral data and thus reconstruction of virtual monoenergetic images (VMIs). Elements with high atomic numbers such as iodine (*Z* = 53) or gadolinium (*Z* = 64) pose a specific energy (measured in keV) at which attenuation is more pronounced than at energies below or above, called the k-edge [12, 13]. VMIs reconstructed at specific energies can help increase contrast-to-noise ratio (CNR) and contrast attenuation (*e.g.*, low keV for iodine) or reduce artifacts, typically from metal objects (*e.g.*, high keV) [14].

The purpose of this study was to evaluate the utility of GBCA as an alternative to ICM for CCTA on a first-generation clinical dual-source PCD-CT system using a dynamic phantom.

## Methods

### Phantom design

**Fig. 1:**
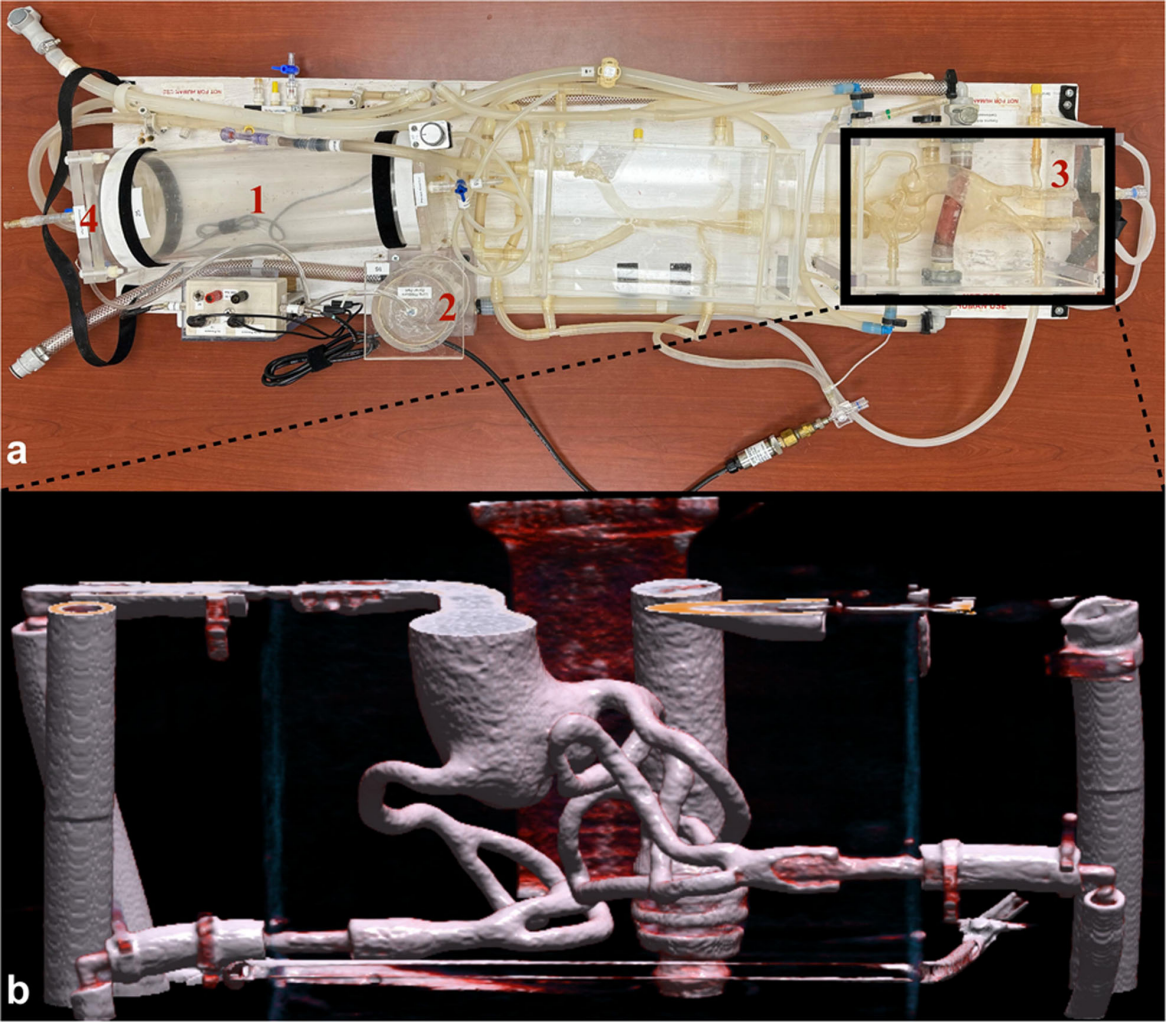
The phantom (**a**) consisted of a low-pressure chamber (1) to mimic pulmonary circulation and a high-pressure chamber for body circulation (2), a cardiac compartment (3), and a pump linked to a circuit board via a connector (4) to imitate a beating heart. **b** A three-dimensional volume render of the cardiac compartment consisting of the ascending aorta and coronary arteries

A custom-built phantom with dynamic circulation was used for this study as previously described [15]. In brief, the phantom consisted of plastic tubing connecting two high-pressure and low-pressure compartments to simulate physiological hemodynamic parameters. Vessels were constructed out of silicone to model the coronary arteries and thoracic aorta (Model T-S-N-002; Elastrat). An acrylic container filled with water was used to encase the vessels and mimic mediastinal attenuation characteristics. The phantom was filled with 4 L of heated water at 37 °C and circulated throughout the system using a modified pulsatile pump to simulate a beating heart (BS4, Harvard Apparatus). An electrocardiography (ECG) simulator was connected to the pump and the CT scanner for real-time synchronization. A heart rate of 68 beats per minute with a stroke volume of 90 mL and a blood pressure of 120/80 mmHg was used to simulate normal physiology. A GBCA (Magnevist®, gadopentetate dimeglumine, Bayer Healthcare) was injected through a dedicated injection port at a rate of 3 mL/s for doses at 50% (0.15 mmol/kg) and 100% (0.3 mmol/kg) the maximum dose of Gd, and at 4 mL/s for doses at 150% (0.45 mmol/kg) and 200% (0.6 mmol/kg), followed by a saline flush at the same injection rate. The recommended dose of gadopentetate dimeglumine for human use is 0.1 mmol/kg while the maximum clinically approved dosage is 0.3 mmol/kg [16]. Details of the contrast injection protocol are listed in Table 1. A three-dimensional render of the model is presented in Fig. 1.

**Table 1:** Contrast injection parameters at different GBCA dose levels

	GBCA dose relative to the clinically approved dose			
	50%	100%	150%	200%
Gd concentration (mL/kg)	0.3	0.6	0.9	1.2
Gd concentration (mmol/kg)	0.15	0.3	0.45	0.6
Gd volume (mL)	15	30	45	60
Single dose equivalent	× 1.5	× 3	× 4.5	× 6
Saline mix (%)	50	0	0	0
Saline flush volume (mL)	30	30	30	30
Trigger HU above baseline	50	50	50	50

*GBCA* Gadolinium-based contrast agent, *Gd* Gadolinium

### Data acquisition

All acquisitions were performed on a first-generation dual-source PCD-CT system (NAEOTOM Alpha, Siemens Healthineers) with simulated ECG triggering at 60% of the RR interval. The PCD-CT system contains two cadmium telluride detectors with a collimation of 144 × 0.4 mm on each detector facilitating the acquisition of spectral data with a high temporal resolution. Automatic dose modulation was turned off to minimize variability in radiation dose between the applied contrast concentrations.

A region of interest was placed in the descending aorta to allow for bolus tracking with the scan triggered at 8 s after reaching specific attenuations listed in Table 1. The system was completely drained and flushed with water after each scan to avoid contamination from previous scans. Before each new scan, a flush scan with water was performed to rule out contamination. Data acquisition was performed at 120 kVp with 25 mAs to produce a CT dose index volume−CTDI_vol_ of 12.8 mGy for contrast-enhanced scans and 7.73 mGy for the flush scans. Total injection times were 15 s for the 50%, 20 s for the 100%, 19 s for the 150%, and 23 s for the 200% doses.

### Image reconstruction

Reconstruction of VMIs was performed directly on the scanner front end in 5 keV increments ranging from 40 keV to 100 keV for a total of 13 reconstructions per GBCA concentration for a total of 52 datasets for the coronary arteries and aorta each. Slice thickness and increments were set at 0.6 mm and 0.5 mm, respectively. A soft/medium reconstruction kernel (Qr36) with a quantum iterative reconstruction strength level of 2 was used [17].

### Image analysis

A board-certified radiologist (C.G.) with 4 years of cardiovascular imaging experience analyzed each reconstruction. Vessel attenuation was measured in HU by placing the largest possible region of interest in the following vessels: ascending aorta, descending aorta, left coronary artery, left anterior descending, left circumflex, and the right coronary artery. Additionally, measurements at the left anterior descending, left circumflex, and right coronary arteries were performed at proximal, middle, and distal vessel segments.

Noise was defined as the standard deviation of a 1 cm^2^ region of interest placed in the water tank at the level of the left coronary artery, and CNR was calculated as previously described [18]. Diagnostic attenuation of the coronary arteries was defined as HU values of at least 350 HU, as per current recommendations [19]. Attenuation, noise, and CNR values of a polychromatic reconstruction from a previous study using iodine as a contrast medium performed on the same scanner and phantom with identical settings were used as a reference standard for comparison [15].

### Statistical analysis

Statistical analysis was performed using SPSS v29 (IBM Corporation) and GraphPad Prism Version 10.2.1 (GraphPad Software). The Shapiro–Wilk test was used to test continuous data for normality. Normally distributed variables are reported as means ± standard deviation. Nonnormal distributions are reported as median with interquartile range. Categorical variables are reported as absolute frequencies and proportions. Levene’s test for equality of variance was used to assess data, if negative, one-way analysis of variance—ANOVA with Tukey’s post hoc tests was used to assess differences of means for attenuation and CNR, if positive, differences were evaluated using the Friedman test. Values of *p* < 0.05 were considered significant.

## Results

**Fig. 2:**
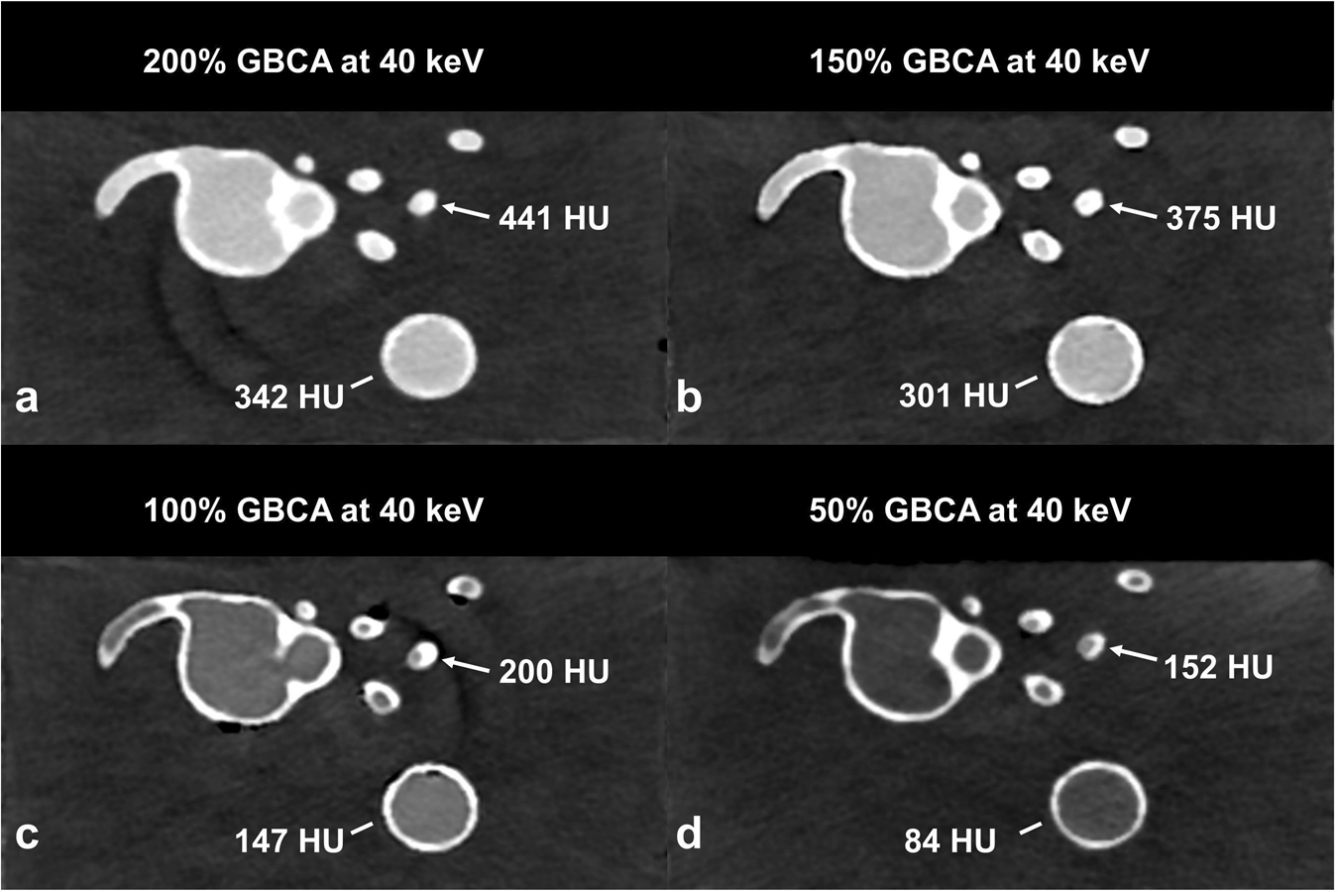
Axial slice of the model at the origin of the right coronary artery, at 40 keV and different concentrations of gadolinium (**a**–**d**) demonstrating differences in attenuation values (HU) of the coronaries (arrows) and the aorta (lines)

A systematic decline in attenuation and CNR was observed going from higher to lower concentrations of GBCA, as well as from lower to higher keV levels. Sufficient diagnostic attenuation of the coronary arteries was only achieved at concentrations of 150% and 200% of the maximum GBCA dose at 40 keV (HU_150%_ 375 ± 42 and HU_200%_ 441 ± 23) and at 200% maximum GBCA at 45 keV (361 ± 18 HU). A significant difference in coronary attenuation was observed among all keV levels at concentrations above 50% maximum GBCA dose (*p* ≤ 0.0003). Some comparisons at 50% maximum GBCA dose did not demonstrate significant differences in coronary attenuation, *e.g.*, at 40 keV *versus* 45 keV (*p* = 0.344) or at 40 keV *versus* 50 keV (*p* = 0.089). Detailed comparisons are provided in Table 2. A visual representation of attenuation values of the model at the same keV but at different GBCA concentrations is shown in Fig. 2.

**Table 2:** Coronary artery attenuation at different keV levels and GBCA concentrations

	Attenuation, (HU)				
Reconstructions	Iodine^a^	GBCA_50%_	GBCA_100%_	GBCA_150%_	GBCA_200%_
T3D	482 ± 10				
40 keV	1,253 ± 38	152 ± 66	200 ± 46	**375** ± **42**	**441** ± **23**
45 keV	1,008 ± 28	113 ± 48	164 ± 37	306 ± 34	**361** ± **18**
50 keV	824 ± 10	95 ± 37	135 ± 30	252 ± 28	298 ± 14
55 keV	680 ± 5	73 ± 26	113 ± 25	209 ± 24	248 ± 11
60 keV	565 ± 5	67 ± 28	95 ± 21	176 ± 21	210 ± 9
65 keV	473 ± 7	48 ± 19	81 ± 19	150 ± 19	181 ± 8
70 keV	398 ± 9	41 ± 20	72 ± 17	132 ± 18	158 ± 8
75 keV		36 ± 18	64 ± 16	117 ± 16	140 ± 7
80 keV		31 ± 19	58 ± 16	105 ± 15	125 ± 7
85 keV		27 ± 16	53 ± 14	94 ± 14	113 ± 7
90 keV		17 ± 13	48 ± 14	86 ± 13	103 ± 6
95 keV		15 ± 11	45 ± 14	79 ± 13	94 ± 6
100 keV		12 ± 11	41 ± 13	73 ± 13	87 ± 6

Diagnostic attenuation levels are in bold

*GBCA* Gadolinium-based contrast agent, *T3D* Polychromatic reconstruction

^a^ Iodine findings listed based on the investigation by Emrich et al [15]

**Fig. 3:**
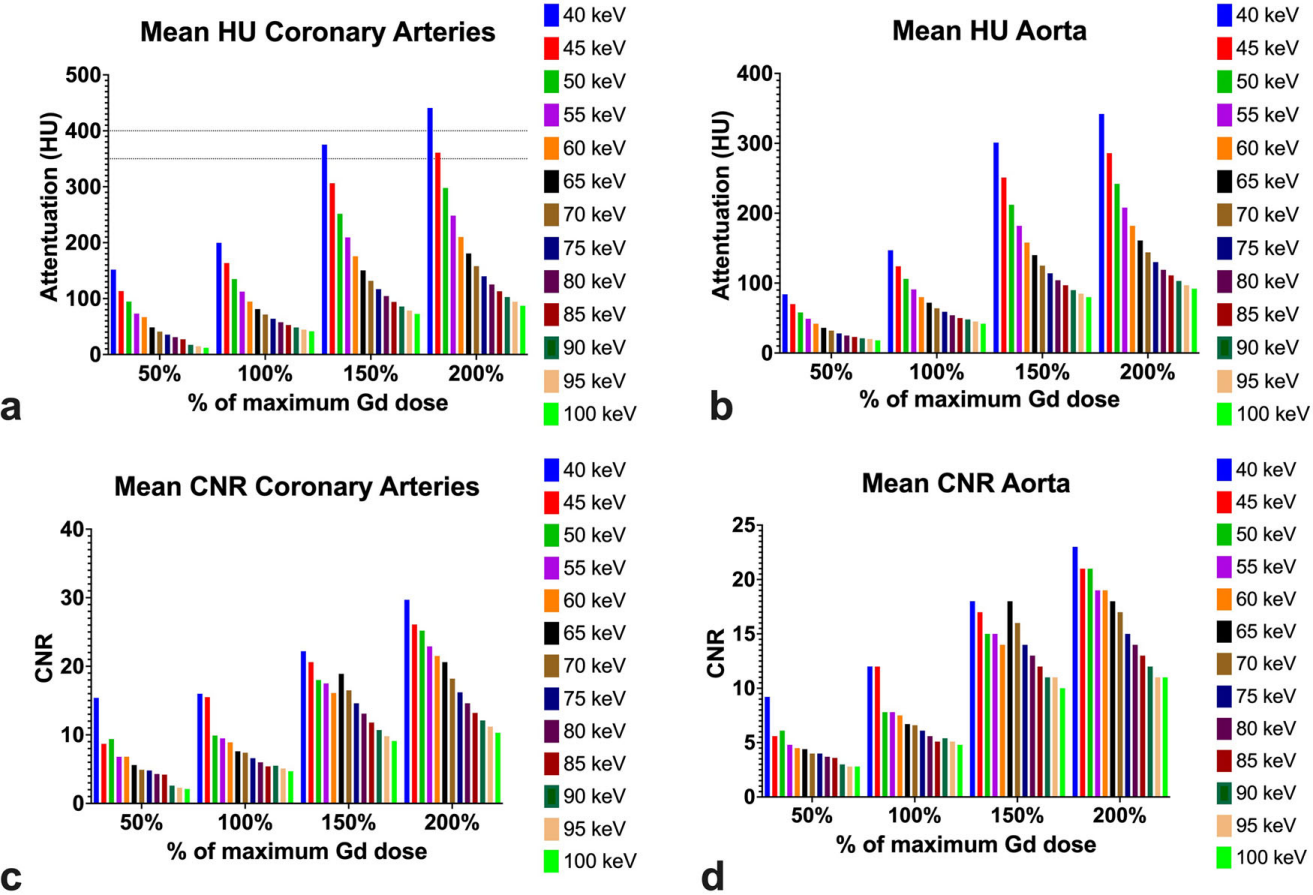
Mean attenuation (HU) for the coronary arteries and aorta (**a**, **b**) and contrast to noise ratios (CNR) (**c**, **d**). Both attenuation and CNR improved with increasing doses of GBCA, although minimum diagnostic attenuation of the coronary arteries defined as HU between 350 and 400 (dotted horizontal lines in (**a**)) was only reached after 150% of the maximum allowable dose. CNR remained below 30 for all vessels at all concentrations

The highest levels of CNR were achieved at 40 keV with 200% of the maximum GBCA dose (29.5 ± 1.5) for the coronary arteries, with the lowest CNR recorded at 100 keV at 50% GBCA (2.1 ± 1.3). A significantly higher CNR was usually achieved at higher GBCA concentrations, *e.g.*, at 40 VMI CNR_50%_ 14.7 ± 5.9 *versus* CNR_200%_ 29.5 ± 1.5 (*p* < 0.0001). Additionally, significantly higher CNR values were noted at lower keVs, *e.g.*, at 150%: CNR_40keV_ 22.3 ± 2.6 *versus* CNR_45keV_ 20.6 ± 2.4 *versus* CNR_50keV_ 18.1 ± 2.1 *versus* CNR_55keV_ 17.5 ± 2.1, all *p* < 0.0001. Visual representations of measurements are provided in Fig. 3. Attenuation and CNR of the coronary arteries and of the aorta are provided in Tables 3 and 4, respectively. Attenuation over 350 HU for the aorta was not achieved in any combination of concentrations and keV. Attenuation above 150 HU was only achieved at GBCA concentrations of 150% (40 keV [301 ± 0.7 HU], 45 keV [251 ± 1.4 HU], 50 keV [212 ± 1.4 HU], 55 keV [182 ± 2.1 HU], 60 keV [158 ± 1.4 HU]), or 200% GBCA (between 40 keV [342 ± 5.7 HU] and 65 keV [161 ± 6.4 HU]).

**Table 3:** Coronary artery contrast to noise ratios at different keV levels and GBCA concentrations

	CNR				
Reconstructions	Iodine^a^	GBCA_50%_	GBCA_100%_	GBCA_150%_	GBCA_200%_
T3D	45 ± 3	−	−	−	−
40 keV	65 ± 8	14.8 ± 5.9	15.8 ± 3.7	22.3 ± 2.6	29.5 ± 1.5
45 keV	57 ± 8	8.4 ± 3.5	15.3 ± 3.5	20.6 ± 2.4	25.9 ± 1.3
50 keV	53 ± 10	9.4 ± 3.6	9.7 ± 2.2	18.1 ± 2.1	25.0 ± 1.2
55 keV	48 ± 12	6.7 ± 2.3	9.4 ± 2.2	17.5 ± 2.1	22.9 ± 1.0
60 keV	41 ± 7	6.8 ± 2.7	8.8 ± 2.0	16.0 ± 2.0	21.4 ± 0.9
65 keV	40 ± 7	5.5 ± 2.0	7.5 ± 1.8	18.8 ± 2.6	20.5 ± 0.9
70 keV	34 ± 3	4.9 ± 2.1	7.3 ± 1.9	16.4 ± 2.3	18.1 ± 0.8
75 keV	−	4.9 ± 2.2	6.6 ± 1.7	14.5 ± 2.1	16.1 ± 0.7
80 keV	−	4.3 ± 2.2	6.0 ± 1.6	12.9 ± 1.9	14.4 ± 0.7
85 keV	−	4.3 ± 2.1	5.4 ± 1.5	11.7 ± 1.8	13.1 ± 0.7
90 keV	−	2.6 ± 1.6	5.5 ± 1.7	10.6 ± 1.7	12.0 ± 0.6
95 keV	−	2.3 ± 1.3	5.1 ± 1.6	9.7 ± 1.7	11.0 ± 0.6
100 keV	−	2.1 ± 1.3	4.7 ± 1.6	8.9 ± 1.6	10.2 ± 0.6

*CNR* Contrast-to-noise ratio, *GBCA* Gadolinium-based contrast agent, *T3D* Polychromatic reconstruction

^a^ Iodine findings listed, courtesy of Emrich et al [15]

**Table 4:** Aorta attenuations and contrast to noise ratios at different keV levels and GBCA concentrations

		HU/CNR			
Reconstructions	Variable	GBCA_50%_	GBCA_100%_	GBCA_150%_	GBCA_200%_
40 keV	HU	84 ± 23	147 ± 15	301 ± 0.7	342 ± 5.7
	CNR	9.2 ± 2.1	12 ± 1.1	18 ± 0.1	23 ± 0.4
45 keV	HU	70 ± 20	124 ± 12	251 ± 1.4	286 ± 5.7
	CNR	5.6 ± 1.4	12 ± 1.1	17 ± 0.1	21 ± 0.4
50 keV	HU	58 ± 16	106 ± 11	212 ± 1.4	242 ± 5.7
	CNR	6.1 ± 1.4	7.8 ± 0.8	15 ± 0.1	21 ± 0.5
55 keV	HU	49 ± 13	91 ± 9.9	182 ± 2.1	208 ± 5.7
	CNR	4.8 ± 1.1	7.8 ± 0.8	15 ± 0.2	19 ± 0.5
60 keV	HU	42 ± 11	80 ± 8.5	158 ± 1.4	182 ± 6.4
	CNR	4.5 ± 1.0	7.5 ± 0.8	14 ± 0.1	19 ± 0.6
65 keV	HU	36 ± 9.2	72 ± 7.8	140 ± 1.4	161 ± 6.4
	CNR	4.4 ± 0.9	6.7 ± 0.7	18 ± 0.2	18 ± 0.7
70 keV	HU	32 ± 7.8	64 ± 7.1	125 ± 1.4	144 ± 6.4
	CNR	4.0 ± 0.8	6.6 ± 0.7	16 ± 0.2	17 ± 0.7
75 keV	HU	28 ± 7.1	59 ± 6.4	114 ± 2.1	130 ± 7.1
	CNR	4.0 ± 0.8	6.1 ± 0.6	14 ± 0.3	15 ± 0.8
80 keV	HU	25 ± 5.7	54 ± 6.4	104 ± 1.4	119 ± 7.1
	CNR	3.7 ± 0.6	5.6 ± 0.6	13 ± 0.2	14 ± 0.8
85 keV	HU	23 ± 5.7	50 ± 5.7	97 ± 2.1	111 ± 7.8
	CNR	3.6 ± 0.7	5.1 ± 0.6	12 ± 0.3	13 ± 0.9
90 keV	HU	21 ± 4.2	48 ± 6.4	90 ± 2.1	103 ± 7.1
	CNR	3.0 ± 0.5	5.4 ± 0.7	11 ± 0.3	12 ± 0.8
95 keV	HU	20 ± 4.9	45 ± 6.4	85 ± 2.1	97 ± 7.1
	CNR	2.8 ± 0.5	5.1 ± 0.7	11 ± 0.3	11 ± 0.8
100 keV	HU	18 ± 4.2	42 ± 5.7	80 ± 2.8	92 ± 7.1
	CNR	2.8 ± 0.5	4.8 ± 0.6	10 ± 0.4	11 ± 0.8

*CNR* Contrast-to-noise ratio, *GBCA* Gadolinium-based contrast agent

## Discussion

This study evaluated the potential use of a clinically approved GBCA for CCTA on a clinical PCD-CT system. The main findings of our study are:

(i) Clinically diagnostic CCTA images were only achievable at concentrations 1.5–2 times the maximum allowable dose of GBCA currently approved for human use.

(ii) As expected with current non-gadolinium-optimized PCD-CT configurations, no discernable k-edge around 50 keV was detected.

Hence, no recommendation for GBCA concentrations for clinical CCTA use can be inferred based on this phantom experiment.

Diagnostic CCTAs are becoming ever more frequent in clinical practice. The assessment of blood vessels requires intravenous injections of ICM, but these can pose certain risks such as contrast-induced nephropathy, thyrotoxic crisis, or anaphylactic shock in patients with hyperthyroidism, iodine allergies, or impaired renal function [20]. Additionally, the use of multi-contrast material decomposition, as demonstrated in an animal study by Symons et al, may eliminate the need for multiphase single-contrast acquisitions [5]. Undoubtedly, there is a potential clinical need to use noniodinated contrast media in CT. GBCAs have already been approved for clinical use in magnetic resonance examinations, have been tested in CT imaging, and offer a favorable safety profile [21].

Equimolar concentrations of Gd produce more attenuation than iodine due to its higher atomic number (*Z* = 64 *versus* iodine *Z* = 53) [6]. However, to achieve higher attenuations on standard energy-integrating-detector CT, higher than clinically acceptable doses of GBCA are required. While spectral imaging has the potential to apply material decomposition and detect lower amounts of GBCA, previous DECT, and spectral CT studies demonstrated low CNRs and suboptimal attenuation of target structures at clinically acceptable concentrations of GBCAs [7, 22]. Similarly, as demonstrated by the present study, even the best result of 200% GBCA dose at 40 keV was not able to match the CNR reference of a polychromatic reconstruction using ICM in the same phantom model and set-up (CNR: ICM 45 ± 3 *versus* GBCA 29.5 ± 1.5) [15]. This might be partly explained by the molecular structure GBCA, where one chelator binds only one active atom of Gd compared to ICM, where one chelator binds three active iodine atoms, so that in turn lower concentrations of contrast media are required to achieve the same effects [23, 24].

A phantom study by Bongers et al on a DECT system yielded the best results for attenuation for GBCAs at 40 keV (mean 616 HU, 0.5 mmol/kg of gadobutrol) [7]. Both, the previous and our present studies fall outside the manufacturer’s recommended GBCA dose for human imaging. Similar findings were also reported in an *in vivo* DECT imaging study of the pulmonary arteries by Xie et al, where the highest attenuations were achieved at 40 keV instead of the expected 50 keV [9].

Baubeta et al demonstrated barely achievable clinically relevant vascular attenuation on PCD-CT using GBCA concentrations approved for human use with no observable k-edge [25]. While Baubeta et al achieved better attenuations at 25 mmol/L concentrations of GBCA (470 ± 16 HU), a true comparison is challenging due to the difference in models used: Baubeta et al used rods approximately 1.3 cm in diameter, while we chose replicas of the coronary arteries, around 0.3 mm in diameter. Additionally, contrasting their static model, we employed a dynamic circulatory model with ECG synchronization and bolus tracking to mimic real-life conditions as much as possible.

The theoretical k-edge of 50.2 keV for Gd has so far not been observed on either DECT or PCD-CT systems as expected, as current systems are not optimized for this detection [26]. A key reason for the lack of observable gadolinium k-edge on PCD-CT and DECT systems involves the configuration for energy binning, which on the user end, is not optimized even for k-edge ICM-based imaging. It should be noted that with a planned future software upgrade, a 4-threshold imaging mode will be made available, together with a multimaterial decomposition algorithm. This may offer additional research opportunities to improve the detection of GBCA and should form the topic of future research in this area.

Due to the current challenges with gadolinium-optimized imaging, achieving the diagnostic image quality of the coronary arteries, where attenuations of > 350 HU are required, remains unfeasible for now, especially at clinically acceptable GBCA doses [19]. Contrary to Gd-based CCTA, recent studies have successfully demonstrated the feasibility of using GBCA for the imaging of larger vessels such as the thoracic [27] and the abdominal aorta [28]. Similarly to the latter, the current study showed comparative attenuations of the aorta at clinically acceptable GBCA doses (0.3 mmol/kg gadopentetate dimeglumine; 147 ± 15 HU at 40 keV), potentially allowing an alternative to ICMs for the CT imaging of large vessels, such as to visualize and follow-up aortic aneurysms, where lower concentrations of GBCAs are acceptable.

The use of GBCAs in CT imaging might have another application in dual contrast imaging. For example, the differentiation between calcium and iodine is worse than in elements with higher atomic numbers, *e.g.*, tungsten, thus potentially allowing improved coronary plaque characterization and better vessel visualization [29]. Dual contrast imaging has also been shown to aid in tissue characterization of the myocardium by combining first-pass iodine and late Gd maps leading to an accurate separation of the blood pool, scar tissue, and myocardium [30]. Another combination of ICM and GBCAs was demonstrated in a recent animal study, potentially allowing for a decreased radiation dose while providing more clinical information [5, 31]. However, as with previous studies, the concentrations of applied GBCA had to be increased beyond the standard clinical doses.

This study possesses the following limitations. While a dynamic circulation phantom was used to mimic a human circulatory system, the model was based on a normal-weight patient and does not consider factors such as obesity and the effects that body composition can have on image quality. Thus, results are not generalizable to the population. All analyses were performed using a single kernel and quantum iterative reconstruction level combination with commercially available settings on the PCD-CT system. Further studies exploring the use of different reconstruction and binning settings are needed to explore the utility of Gd-based PCD-CT imaging. Furthermore, our study analyzed only one GBCA, which is not widely used for cardiac MR imaging. Lastly, the phantom allowed only the assessment of vessel attenuation, therefore the evaluation of plaques or stenoses is the subject of future investigations. Further, *in vivo* studies are required to fully explore the use of GBCAs for PCD-CT coronary imaging.

In conclusion, current PCD-CT settings are unsuitable for the use of Gd for CCTA at clinically approved doses.

## Data Availability

The datasets generated and/or analyzed during the current study are not publicly available but are available from the corresponding author on reasonable request.

## Abbreviations

CCTA: Coronary computed tomography angiography
CNR: Contrast-to-noise ratio
CT: Computed tomography
DECT: Dual-energy computed tomography
ECG: Electrocardiography
GBCA: Gadolinium-based contrast agent
ICM: Iodinated contrast media
PCD-CT: Photon-counting-detector computed tomography
VMI: Virtual monoenergetic image
